# Using the Voices of Transgender, Non-Binary and Intersex Older Adults to Enhance Gerontology/Geriatrics Education

**DOI:** 10.1093/geroni/igaf122.1788

**Published:** 2025-12-31

**Authors:** Nik Lampe, Alexandra “Xan” Nowakowski

**Affiliations:** University of South Florida, Tampa, Florida, United States; Florida State University College of Medicine, Tallahassee, Florida, United States

## Abstract

In this presentation, we utilize semi-structured interview data from transgender, non-binary, and intersex (TNBI) older adults (N = 50) to enrich gerontology and geriatrics education. By incorporating real-world narratives, educators can provide students and professionals with a deeper understanding of the healthcare needs, systemic and interpersonal barriers, and resilience strategies of TNBI older adults. This presentation aligns with the 2025 AGHE Presidential Symposium’s goal of advancing LGBTQIA+ aging education by showcasing applied research and pedagogical strategies that promote critical discussions on researching and delivering evidence-based care. Attendees will gain practical tools for integrating case studies into curricula, ensuring a more inclusive, research-informed approach to addressing the needs and experiences of TNBI older adults.

